# Heterogeneity among Homologs of Cutinase-Like Protein Cut5 in Mycobacteria

**DOI:** 10.1371/journal.pone.0133186

**Published:** 2015-07-15

**Authors:** Deepshikha Verma, Lahari Das, Vandana Gambhir, Kanak Lata Dikshit, Grish C. Varshney

**Affiliations:** Cell biology and Immunology Division, CSIR-Institute of Microbial Technology, Chandigarh-, India; Centre National de la Recherche Scientifique, FRANCE

## Abstract

The study of genomic variability within various pathogenic and non-pathogenic strains of mycobacteria provides insight into their evolution and pathogenesis. The mycobacterial genome encodes seven cutinase-like proteins and each one of these exhibit distinct characteristics. We describe the presence of Cut5, a member of the cutinase family, in mycobacteria and the existence of a unique genomic arrangement in the *cut5* gene of *M*. *tuberculosis* (*Mtb*) strains. A single nucleotide (T) insertion is observed in the *cut5* gene, which is specific for *Mtb* strains. Using *in silico* analysis and RT-PCR, we demonstrate the transcription of *Rv3724/cut5* as *Rv3724a/cut5a* and *Rv3724b/cut5b* in *Mtb* H37Rv and as full length *cut5* in *M*. *bovis*. Cut5b protein of *Mtb* H37Rv (*Mtb*Cut5b) was found to be antigenically similar to its homologs in *M*. *bovis* and *M*. *smegmatis*, without any observed cross-reactivity with other *Mtb* cutinases. Also, the presence of Cut5b in *Mtb* and its homologs in *M*. *bovis* and *M*. *smegmatis* were confirmed by western blotting using antibodies raised against recombinant Cut5b. In *Mtb* H37Rv, Cut5b was found to be localized in the cell wall, cytosol and membrane fractions. We also report the vast prevalence of Cut5 homologs in pathogenic and non pathogenic species of mycobacteria. *In silico* analysis revealed that this protein has three possible organizations in mycobacteria. Also, a single nucleotide (T) insertion in *Mtb* strains and varied genomic arrangements within mycobacterial species make Rv3724/Cut5 a potential candidate that can be exploited as a biomarker in *Mtb* infection.

## Introduction

Members of genus *Mycobacterium* are known to cause deadly diseases like tuberculosis (TB), leprosy and skin ulcers. Among these, TB is a major killer causing death of 2–3 million people per year. According to the WHO global tuberculosis report, 2013 (http://www.who.int/iris/bitstream/10665/91355/1/9789241564656_eng.pdf) the major limitation in TB control is the lack of rapid diagnostics owing to the delayed progress in biomarker discovery. Researchers have exploited the potential of mycobacterial cell wall proteins, secretory proteins, lipoproteins and enzymes, especially those involved in lipid metabolism pathways, in designing novel biomarkers for TB [1–4]. Various growth phase dependent antigens of mycobacteria have also been considered for biomarker development [2, 5].

Despite the fact that *M*. *tuberculosis* (*Mtb*) does not encounter cutin or any of its homologs amid its pathogenic life cycle or environments inside the host, the whole genomic sequence of *Mtb* H37Rv unraveled seven cutinase genes namely *cut1/clp5/Rv1758*, *cut2/clp2/Rv2301*, *cut3/clp3/Rv3451*, *cut4/clp4/Rv3452*, *cut5/clp7/Rv3724*, *cut6/clp6/Rv3802c cut7/clp1/cfp21/Rv1984* [6]. It is likely that these cutinases are involved in alternate functions. These cutinase proteins have already been identified, expressed and discussed in terms of various metabolic pathways and physiological functions in mycobacteria [7, 8].

Cutinases are present in both environmental and pathogenic strains of mycobacteria [9, 10]. Cutinases are α/β hydrolases, which possess a conventional catalytic triad with a serine residue located within the conserved pentapeptide G-X-S-X-G motif. Gamieldein et al., 2002 [11] proposed 19 genes from the cutinase family in *Mtb* which may have been acquired from eukaryotes during evolution. Four mycobacterial cutinases, namely *Rv1758*, *Rv3451*, *Rv3452* and *Rv1984c*, were thought to be acquired by horizontal gene transfer, as no bacterial orthologs were found for these mycobacterial cutinases. On the other hand, cutinases of oomycetes which are closely related to diatoms and brown algae have been speculated to be acquired from mycobacteria by lateral gene transfer. The absence of cutin in the mycobacterial environment is evocative of the divergence of mycobacterial cutinases from other genera bearing cutinases [9].

The cutinase motif is prevalent in environmental and pathogenic strains of mycobacteria. Various environmental mycobacterial species such as *Mycobacterium* sp. KMS and *M*. *vanbaalenii* have been reported to contain homologs of cutinase encoding genes [9]. Phylogenetic analysis revealed: i) the presence of gene duplications among other members of mycobacterial cutinases [8], ii) strong bootstrap support for orthology between *cut6* and *M*. *leprae* cutinase and iii) the conserved nature of Cfp21/Cut7 protein in the genome of *Mtb H37*Rv and *M*. *bovis* (*Mbovis*_2006c), although this protein is deleted from most of the strains of BCG.

In spite of the availability of genome data of *Mtb*, and some other mycobacteria, the presence of cutinases in mycobacteria remained unknown for a decade until Parker et al. 2007 purified and characterized the phospholipase A (Cut4) from *M*. *smegmatis* culture supernatant and associated this activity with the putative mycobacterial cutinase. Cut2 and Cut7 are secreted proteins that have been found as components of culture filtrate [12], whereas Cut6 has been shown localized in the cell wall of *Mtb* and belongs to the gene cluster which is specifically found to encode proteins involved in mycolic acid synthesis [7]. With a predicted molecular weight of 23 kDa, Cut2 has previously been named Cfp23. Parker et al. 2007 demonstrated that Cut4 is secreted in the culture supernatant of *M*. *smegmatis* and resides within the cell wall of *Mtb*.

Among all members of the mycobacterial cutinase family, Cut2 and Cut7 were found to be highly immunogenic and protective as protein vaccines [13, 14]. High titer antisera were obtained for Cut2, Cut3 and Cut7/Cfp21. Evaluation of a serological response along with immunoblot analysis of Cut2, Cut3, Cut6 and Cut7, with antiserum raised against the individual proteins, indicated the lack of cross-reactivity [14].

The secreted cutinases like Cut7 and Cut4 have also been reported to have potential as biomarkers in patients with active tuberculosis, thereby making these cutinases suitable candidates for the production of a TB vaccine [1, 14]. Transcripts of *Rv3451/cut3* and *Rv3724/cut5a* have been shown to be elevated during *in vivo* survival of mycobacteria inside hypoxic foamy macrophages [15]. Recently, Rv3451/Cut3 has been reported as the primary trehalose dimycolate hydrolase in *Mtb*. It is induced in nutrient limiting conditions which helps in nutrient influx and simultaneously sensitizes the bacteria towards intracellular stresses encountered in the host [16]. Also, the role of Cut2 in host cell invasion has been reported, making it a potential vaccine candidate [17]. All of these reports emphasize the relevance of the cutinase family of proteins in *Mtb* pathogenesis.

*Rv3724* in *Mtb* encodes two different protein products, Cut5a and Cut5b, while in other mycobacterial species, the *cut5* gene is transcribed as a single product. Here, we examined: i) the evolution of the *cut5* gene at the nucleotide and protein levels, ii) antigenic similarity and cross reactivity among Cut5 homologs in mycobacteria, iii) subcellular localization of Cut5b in *Mtb* H37Rv and iv) N-terminal sequences of Cut5 and its homologs in various mycobacterial species.

## Materials and Methods

The plasmid construct pET 19b-cut1 and antisera generated against Cut2, Cut6 and Cut7/cfp21 were kind gifts from Dr. Nicholas P. West, Centenary Institute, Sydney, Australia. The following reagent was obtained through the NIH Biodefense and Emerging Infections Research Resource Repository, NIAID, NIH: monoclonal anti-*Mycobacterium tuberculosis* HspX (Gene *Rv2031c*), Clone CS-49 (produced *in vitro*), NR-13814. Genomic DNA of *Mtb* H37Rv was a kind gift from Dr. G P S Raghava and Dr. Ashwani Kumar IMTECH, Chandigarh, India.

### Bacterial growth conditions

*Mtb* H37Rv (originally procured from University of Berkley, California was a kind gift from Dr. Ashwani Kumar, IMTECH, Chandigarh, India), *M*. *smegmatis* mc^2^ 155 (ATCC 607) and *M*. *bovis* BCG (ATCC 35734) were grown in Middlebrook 7H9 broth supplemented with ADC (Difco Laboratories, Detroit, MI) and 0.5% pyruvate (in case of *M*. *bovis* BCG) for 14 days at 37°C. Bacterial cells were plated on Middlebrook 7H11 agar plates supplemented with OADC (Difco Laboratories, Detroit, MI) and were stored in 30% glycerol at -70°C after enumeration.

### Construction of recombinant plasmids, expression and purification of recombinant proteins

All target genes were amplified from *Mtb* H37Rv, *M*. *bovis* or *M*. *smegmatis* genomic DNA using a specific set of forward and reverse primers for each gene that incorporated a 5’ NdeI and a 3’BamHI site to express a protein with a C-terminal histidine tag. The primers used were as follows (F) 5’-GATCCATATGGCACCGGGGAGTCACCTGGTATT-3’ and (R) 5’-GATCGGATCCCTACAGGCGGCTGGCGGCGAATT-3’ for *Rv3724b/cut5b*; (F) 5’-GATTCATATGGATGTCATCAGATGGGCTCG-3’ and (R) 5’-GATCGGATCCCTACAGGCGGCTGGCGGCGAATT-3’ for *Mb_3751/cut5*; (F) 5’–ATATATATCATATGATGAACGTT-3’ and (R) 5’-ATATATGGATCCCAACCGGTC-3’ for *Msmeg_5878*. The PCR products were ligated into pET28c. These constructs were transformed into *E*. *coli* BL21 (DE3). Expression conditions included induction with 1 mM IPTG at O.D_600nm_ 0.4 for 12 hours at 25°C in LB broth containing kanamycin (50μg/ml). Cells overexpressing the proteins were harvested by centrifugation at 2,500g, for 10 min, at 4°C and resuspended in a buffer containing 50 mM NaH_2_PO_4_, 20 mM NaCl, 10 mM imidazole and 2.5 mM CHAPS. Cells were lysed by sonication and the lysate was cleared by centrifugation at 12,000g for 10 min at 4°C. Lysate containing the soluble protein was purified using a Ni-NTA column (Qiagen) according to manufacturer’s instructions. The purity of the protein was checked on a 12% SDS-PAGE gel after coomassie staining. We could purify recombinant Cut5b of *Mtb* H37Rv (*Mtb*rCut5b) and Cut5 of *M*. *bovis* (*Mbov*rCut5). However, in case of *M*. *smegmatis* we could not get the purified recombinant protein. Accordingly, the lysate of *E*. *coli* overexpressing the protein was taken for further studies.

### Animals and Antisera generation

Inbred BALB/c mice (6–8 weeks old) were obtained from the Central animal facility of CSIR-IMTECH, Chandigarh, India. Mice were originally procured from Jackson Laboratory, in Bar Harbor, USA and were reared under conventional conditions with a pellet diet and water *ad libitum*. Protocols involving immunization and sera collection were approved by the Institutional Animal Ethics Committee of Institute of Microbial Technology, Chandigarh, India under the project approval number: IAEC 10/8 and IAEC 13/18. Groups of 5–6 female BALB/c mice, 8–10 weeks old, were immunized with purified protein (25 g in Freund’s complete adjuvant/mouse). Pre-immune sera, which served as control, were first collected by tail bleeding of the mice before immunizations. Mice were immunized subcutaneously (IP/SC) with the above materials and boosters were given in Freund’s incomplete adjuvant at 3 week intervals. After 3–4 boosters, blood was collected by orbital puncture, pooled and kept at 4C for 1–2 hours. After breaking the clot, the blood was centrifuged at 400g at 4C for 10 min. The sera was collected, decomplemented at 56C for 30 min and stored in aliquots at -70C after adding sodium azide to a final concentration of 0.02%.

### Subcellular fractionation of mycobacteria

*M*. *bovis and M*. *smegmatis* were cultured in Middlebrook 7H9 broth at 37°C with mild agitation. Log phase cultures (O.D600_nm_ 0.5–0.6) were harvested by centrifuging at 2,500g for 15 min at 4°C. The pellets were washed three times with PBS. For lysing the cells, the final pellet was resuspended in a breaking buffer (containing 0.1% Tween 80, 1mM MgCl_2_, 1mM benzamidine and a protease inhibitor cocktail [Pierce] in PBS). The bacterial suspension was sonicated (Ultrasonic homogenizer, Cole and Parmer Instruments, Chicago) in an ice bath for 25 minutes followed by centrifugation at 10,000g for 10 min. The supernatant containing the cell free extract was collected and stored at -70°C. For the preparation of the extract and the subcellular fractions of *Mtb* H37Rv, the bacterium was cultured in Middlebrook 7H9 broth containing 10% ADC at 37°C with mild agitation in the Biosafety level 3 facility of the CSIR-IMTECH, Chandigarh, India. Log phase cultures (O.D_600nm_ 0.5–0.6) were harvested by centrifuging at 2,500g for 15 min at 4°C. The pellet was washed three times with PBS. For lysing the cells, the final pellet was resuspended in breaking buffer. One ml of the suspension was added to 0.5ml of 0.1mm glass beads in a screw capped tube with an O- ring and then subjected to 3 × 1 min pulses with a 1 min rest on ice. The lysate was collected by centrifugation at 13,000g for 10 min at 4°C. The supernatant (or the cell free extract) was collected and centrifuged at 27,000g for 20 min at 4°C. The pellet containing cell wall proteins was resuspended in PBS containing 2% SDS and kept overnight at room temperature (RT). For obtaining the cytosolic and membrane fractions, the extract was subjected to ultracentrifugation at 100,000g for 1 hour at 4°C. The supernatant containing the cytosolic fraction was collected and the pellet containing the membrane fraction was resuspended in PBS containing 2% SDS. All fractions were stored at—80°C.

### SDS—PAGE and Immunoblotting

Protein samples and molecular weight standards (BioRad) were separated on a 12% SDS-PAGE gel and transferred to a nitrocellulose membrane following a standard protocol [18]. Blots were probed with an appropriate dilution of polyclonal antisera or monoclonal antibodies. A goat- anti- mouse-HRP (BioRad) secondary antibody was used and a blot was developed with an ECL Plus chemilluminescent substrate (GE healthcare).

### DNA and RNA extraction

Genomic DNA of *Mtb* H37Rv was used for PCR amplification of full length *Rv3724*, *Rv3724a* and *Rv3724b*. For the total RNA extraction, mid—log phase cultures of *Mtb* H37Rv and *M*. *bovis* BCG were harvested by centrifugation at 3000g for 10 minutes at 4°C. The bacterial pellet obtained was washed in a GTC buffer containing 5M guanidium thiocyanate (Sigma-Aldrich), 0.5% Tween-80 (Sigma-Aldrich) and 1% beta mercaptoethanol (Merck). FastRNA Pro (MP biomedicals) was added to the pellet and the RNA extraction was performed according to the manufacturer’s instructions. The RNA obtained was checked for its purity on a 1% non denaturing agarose gel.

### RT-PCR analysis

For the RT-PCR analysis, RNA was extracted from mid—log phase cultures of *Mtb* H37Rv and *M*. *bovis* BCG. Synthesis of cDNA was carried out using a Thermo Scientific First Strand cDNA Synthesis Kit (Cat no.K1611) as per the manufacturer’s instructions. The cDNA obtained was directly used for PCR amplification of *cut5a*, *cut5b* and full length *cut5* using gene specific primers. The amplicons were visualized on a 1% agarose gel.

### Immunoelectron microscopy

For sample preparation, standard protocols were followed with minor modifications [19–22]. Briefly, log phase cells of *Mtb* H37Rv were fixed overnight with in PBS containing 4% paraformaldehyde and 0.2% glutaraldehyde at 4°C. It was then washed twice with 0.1M cacodylate buffer and subjected to dehydration with an ethanol gradient (30%, 50%, 70%, 80% and 90%) for 30 min and absolute ethanol for 1h at 4°C. Embedding was done in LR-white acrylic resin (Sigma-aldrich) for 36h at 55°C. Ultrathin sections (80nm thick) were cut with a Leica EM UC7 ultramicrotome and collected on nickel grids and processed for immunogold labeling. Grids were blocked with PBS containing 0.01% Tween-20 and 2% skimmed milk for 30 min at room temperature followed by three washes in PBST. Primary antibody incubation was done with 1:100 dilution of anti-Cut5b antiserum or pre-immune serum for 16–18 h at 4°C. Grids were washed and incubated with a 1:5 dilution of 10nm gold nanoparticles conjugated goat- anti- mouse secondary antibody (Sigma) for 1h at room temperature. Three washes were performed with PBST and one with water before staining the grids with 2% aqueous uranyl acetate. Samples were then observed under JEOL 2100 TEM.

### Bioinformatics analysis

#### Sequence retrieval

*Rv3724a/Cut5a* and *Rv3724b/Cut5b* sequences of *Mtb* H37Rv and its homologs in *M*. *bovis* and *M*. *smegmatis* were retrieved from a Tuberculist database (genolist.pasteur.fr/tuberculist). Gene sequences of mycobacterial homologs were retrieved from a KEGG genomic database (http://www.genome.jp/kegg/genes)

#### Alignment studies and percentage GC calculation

Nucleotide derived amino acid sequences were compared with a non-redundant database in PSI-BLAST or BLASTP (Basic Local Alignment Tool for protein sequences) program using a mail server at the NCBI website [23]. Gap opening and extension penalties of 10 and 0.02, respectively, were used during the alignments. Sequence alignment was initially performed using the multiple sequence alignment software programs ClustalW (www.ebi.ac.uk/Tools/msa/clustalw2/‎ (and ClustalOmega (www.ebi.ac.uk/Tools/msa/clustalo/‎). The results were displayed with ESPIRIT [24] and adjusted manually. Genomic sequences of *Rv3724a*, *Rv3724b* and their homologs in different mycobacterial species were analyzed for percentage GC content using the online available tool GC Calculator (www.genomicsplace.com/gc_calc.html)

## Results

### *In silico* analysis of *M*. *tuberculosis* H37Rv *cut5* (*Rv3724*)

**Fig 1:**
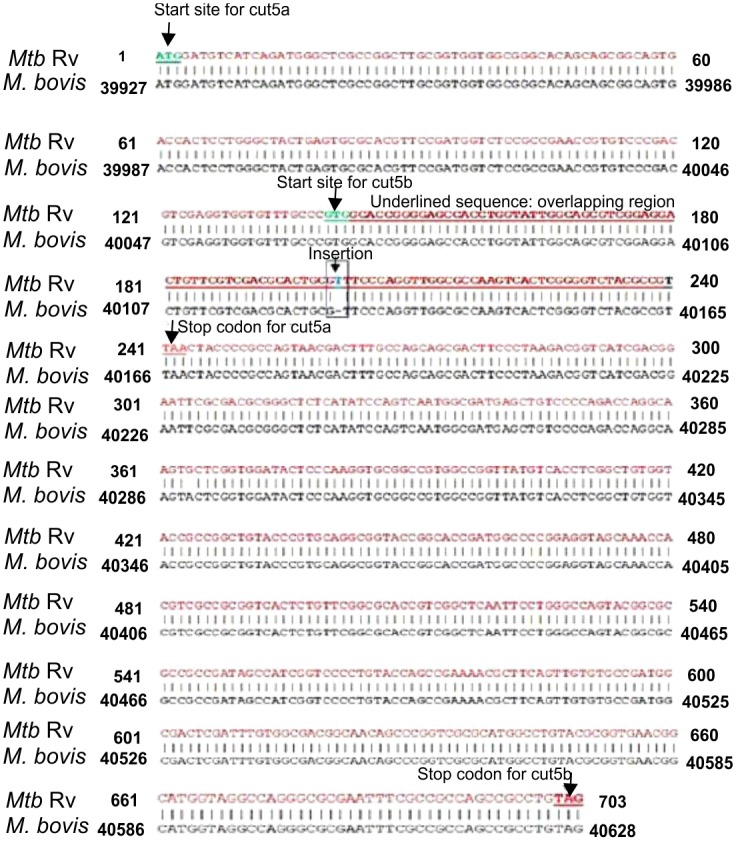
Complete nucleotide sequences of the genes encoding Cut5a and Cut5b (in *M*. *tuberculosis* H37Rv) and Cut5 (in *M*. *bovis*).

NCBI, Tuberculist, tbdb and KEGG are few databases where a single gene *Rv3724* (*cut5*) has been annotated to encode two protein products Cut5a and Cut5b in *Mtb* H37Rv. To understand the rationale behind such an arrangement, the genomic sequences of *Rv3724* and its homologs from different mycobacterial species were studied. Its homolog, *M*. *bovis* (*Mb_37*51) showed 100% identity with *cut5a* and *cut5b*. However, in *Mtb* H37Rv a single nucleotide (T) insertion creates a stop codon within the gene that leads to the translation of Cut5a and Cut5b from different reading frames (Fig 1).

**Fig 2:**
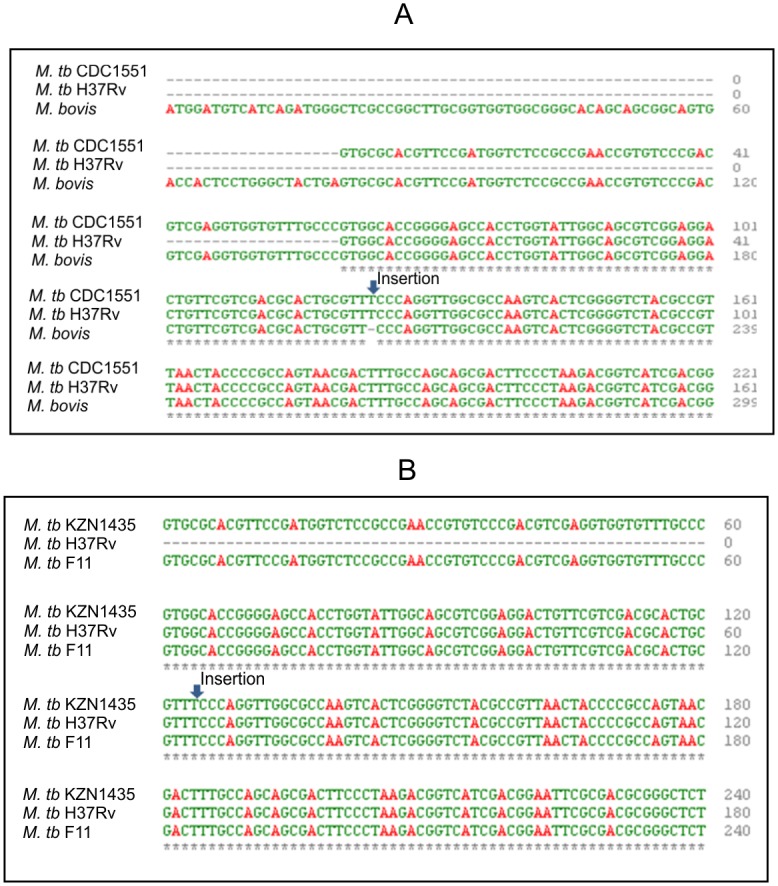
Clustal omega alignment of *Rv3724b* in *Mtb* H37Rv and its homologs.

This single nucleotide (T) insertion is also present in the homologs of *Cut5* in pathogenic *Mtb* strains like CDC1551, KZN1435 and F11 (Fig 2). It is likely that this particular single nucleotide insertion is a unique molecular signature of *Mtb* strains.

### RT-PCR analysis of *cut5* from *M*. *tuberculosis* H37Rv and *M*. *bovis* BCG

**Fig 3:**
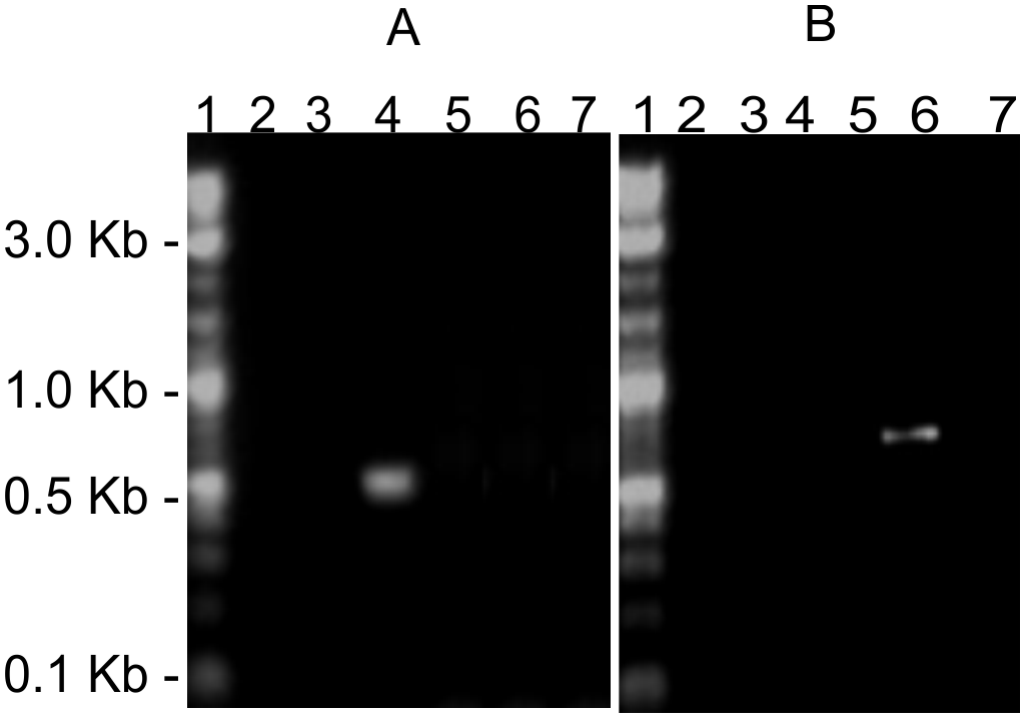
Expression of *Rv3724* and its homolog *Mb_3751* in *Mtb* H37Rv and *M*. *bovis* BCG respectively.

In order to understand the consequence of a mutation on the occurrence of Cut5 in mycobacteria, its expression at the mRNA level was checked in *Mtb* H37Rv and *M*. *bovis* using RT-PCR. The expression of *Rv3724/cut5* gene and its homolog *Mb_3751* were checked with total RNA extracted from a mid log phase culture of *Mtb* H37Rv and *M*. *bovis* BCG respectively. Gene specific primers were used to amplify their respective transcripts. A transcript of *Rv3724b* (confirmed by DNA sequencing) was detected but no transcripts for *Rv3724a* and full length *Rv3724* were obtained from the *Mtb* H37Rv genome (Fig 3, panel A). However, the same primers could amplify *Rv3724a* (243 bp), *Rv3724b* (564 bp) and full length *Rv3724* (702 bp) from the *Mtb* H37Rv genomic DNA (S1 Fig). In case of *M*. *bovis* BCG, only the transcript of full length *Mb_3751* could be detected (Fig 3, panel B). This observation was in accordance with the bioinformatics analysis which indicated that the *Rv3724/cut5* gene is transcribed as *Rv3724a/cut5a* and *Rv3724b/cut5b* only in *Mtb*. The expression of *Rv3724a/cut5a* could not be detected under the given test conditions, although its transcript has been reported to be upregulated in dormant mycobacteria residing in lipid loaded macrophages [15].

### Specificity of Cut5 homologs in mycobacteria

**Fig 4:**
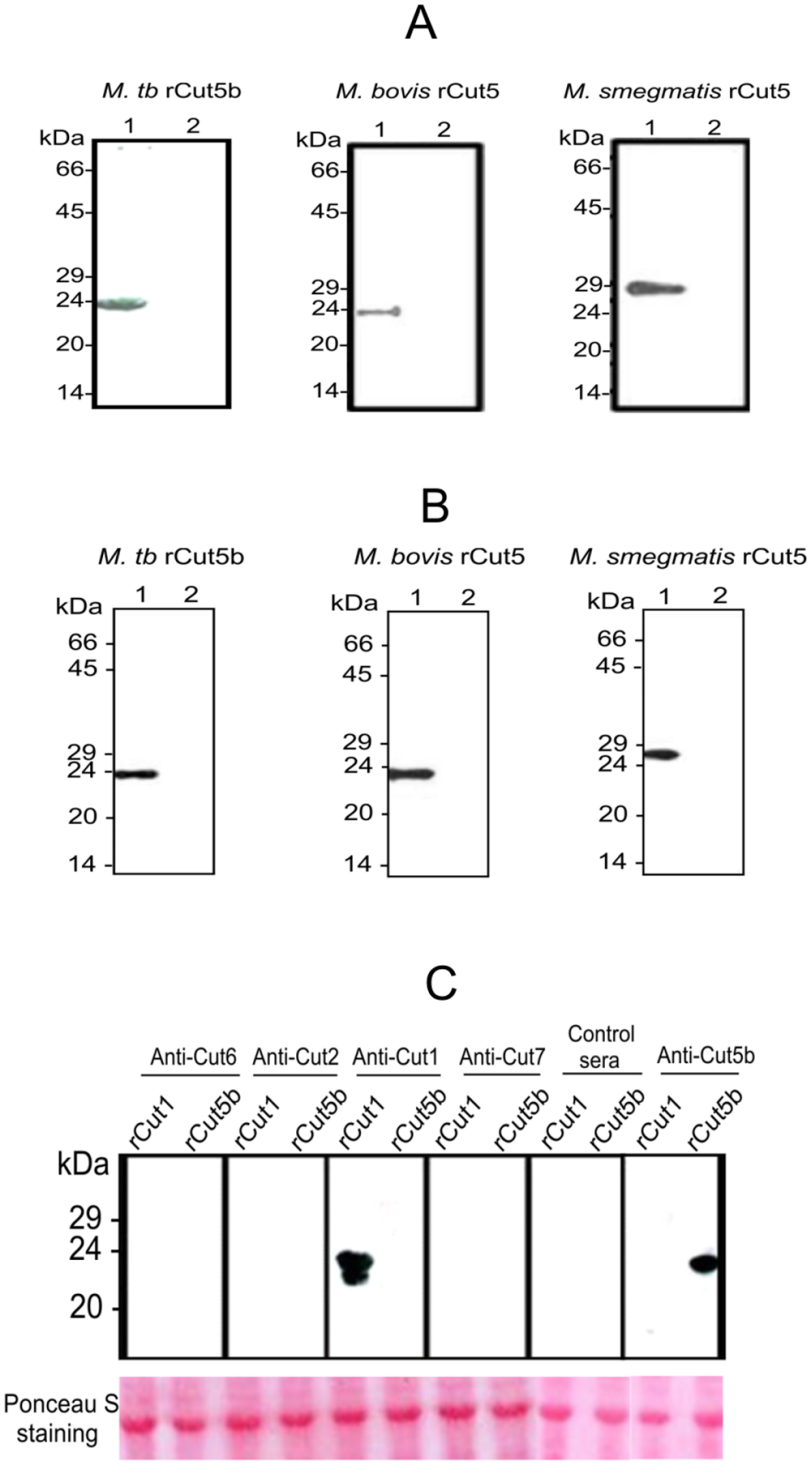
Specificity of Cut5 homologs in mycobacteria.

First, in order to study the antigenic similarities among Cut5 homologs in mycobacterial species recombinant proteins (Cut5b of *Mtb* H37Rv [*Mtb*rCut5b] and Cut5 of *M*. *bovis* [*Mbov*rCut5]) or *E*. *coli* cell lysate containing recombinant Cut5 of *M*. *smegmatis* (*Ms*rCut5) were probed with anti- *Mtb*rCut5b antisera (*Mtb*Cut5b sera) and anti-*Mbov*rCut5antisera (*Mb*Cut5 sera) in western blotting. Both these sera showed good cross reactivity among Cut5 homologs in *Mtb* H37Rv, *M*. *bovis* and *M*. *smegmatis* (Fig 4, panels A & B). These results demonstrated the antigenic similarities among the Cut5 homologs in the mycobacterial species studied.

Next, in order to check whether some antigenic cross-reactivity exists between Cut5 and other cutinases of *Mtb* H37Rv, comprehensive information pertaining to protein sequence homology of different *Mtb* H37Rv cutinases was retrieved from the literature. Table 1 shows that among all cutinases, Cut1 bears a maximum sequence homology (~56%) at protein level with Cut5 while other cutinases namely Cut2, Cut4 and Cut7/Cfp21 share nearly 45–50% homology with Cut5. Accordingly, the possible existence of cross-reactive epitopes among these cutinases was checked by using antisera raised against *Mtb*rCut2, *Mtb*rCut7/Cfp21, *Mtb*rCut6, *Mtb*rCut1 and *Mtb*rCut5b with the lysate of *E*.*coli* cells overexpressing *Mtb*rCut1 and *Mtb*rCut5b in immunoblotting. The results (Fig 4, panel C) indicate that in spite of sequence homology among *Mtb* H37Rv cutinases, there was no antigenic cross-reactivity among Cut5 and other mycobacterial cutinases conferring the specificity of generated antibodies towards Cut5.

**Table 1:** Percentage homology based on amino acid sequences of cutinases of *Mtb*H37Rv.

Nomenclature	Percentage homology among *Mtb*H37Rv cutinases***						
	Cut1	Cut2	Cut3	Cut4	Cut5	Cut6	Cut7
Rv1758*/Cut1*/Clp5**	100						
Rv2301*/Cut2*/Clp2**	39	100					
Rv3451*/Cut3*/Clp3**	36	39	100				
Rv3452*/Cut4*/Clp4**	45	54	63	100			
Rv3724*/Cut5*/Clp7**	56	38	35	46	100		
Rv3802c*/Cut6*/Clp6**	18	22	17	21	27	100	
Rv1984c*/Cut7(Cfp21)*/Clp1**	49	44	49	53	49	20	100

*Tuberculist database (genolist.Pasteur.fr/Tuberculist),

**West *et al*., 2009

***Adapted from West *et al*., 2008

### Presence of Cut5b homologs in mycobacteria and its subcellular localization in *M*. *tuberculosis* H37Rv

**Fig 5:**
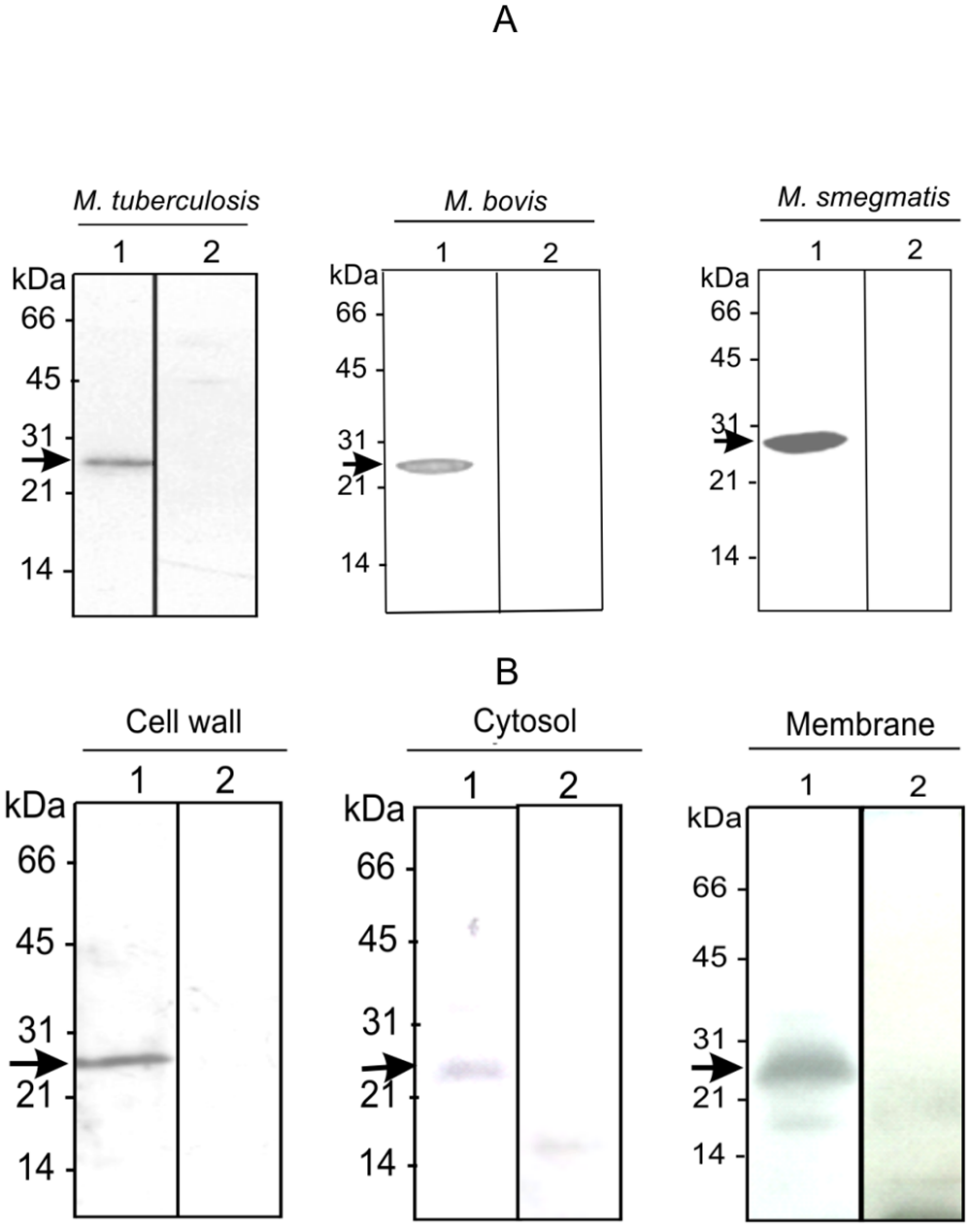
Existence of Cut5b and its homologs in mycobacteria.

**Fig 6:**
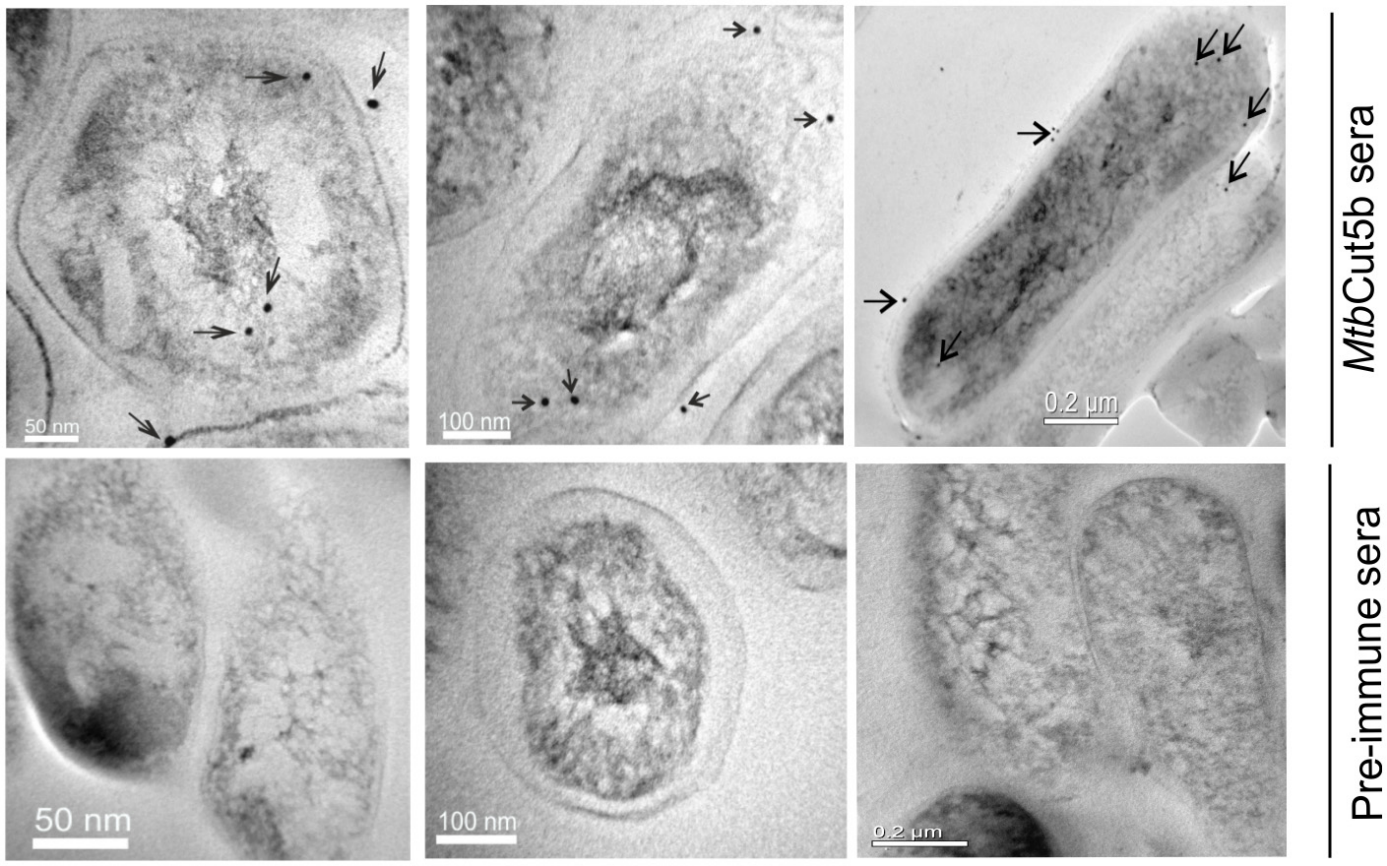
Immunoelectron microscopy showing localization of Cut5b in *Mtb* H37Rv.

The presence of Cut5b and its homologs was demonstrated in the cell free extracts of *Mtb* H37Rv, *M*. *bovis* and *M*. *smegmatis*. *Mtb*Cut5b sera was able to detect Cut5b in *Mtb* H37Rv at ~24kDa and its homologs in *M*. *bovis* and *M*. *smegmatis* at ~24kDa and ~27kDa respectively (Fig 5, panel A). Also, in subcellular fractions (cell wall, cytosol and membrane) of *Mtb* H37Rv, Cut5b was recognized by *Mtb*Cut5b sera at ~24kDa (Fig 5, panel B). *Mtb*Cut6 sera, taken as a positive control, showed the presence of Cut6 at ~34kDa in cell wall and membrane fractions only (data not shown). Further, immunoelectron microscopy results (Fig 6) were consistent with above results and confirmed the presence of Cut5b in *Mtb*.

**Fig 7:**
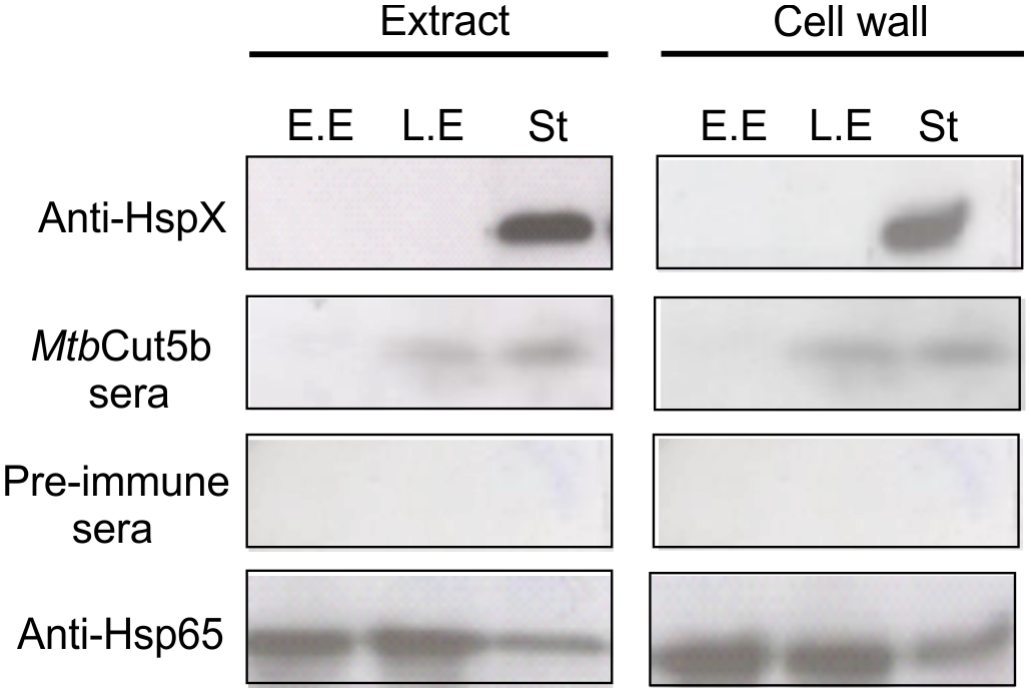
Expression of Cut5b at different *in vitro* growth phases of *Mtb* H37Rv.

Cut6, a cell wall associated cutinase, has been reported to be essential for *in vitro* growth of mycobacteria [8, 25]. Initially the occurrence of Cut5b in different phases of *in vitr*o growth of mycobacteria was investigated. *Mtb* H37Rv cultures were harvested at early exponential (O.D_600_ 0.2), late exponential (O.D_600_ 0.7) and stationary phase (O.D_600_ 1.8). Cell free extracts and the cell wall fractions were prepared, immunoblotted and probed with *Mtb*Cut5b sera. The HspX (16 kDa), a highly abundant protein in the stationary phase of mycobacteria [26], was used as a positive control. Cut5b was found to be present at late exponential and stationary phases and was not detected in the early exponential phase (Fig 7). This observation is consistent with the fact that enzymes like cutinases (e.g. Cut4 and Cut6) can remain associated with cell wall where they may be involved in cell wall biosynthesis, its modification, lipid metabolism and host lipid scavenging that are essential for *in vitro* growth, intracellular replication and adaptation to dormant phases [8, 15, 27].

### Study of *M*. *tuberculosis* H37Rv Cut5a and Cut5b homologs in other mycobacterial species

**Fig 8:**
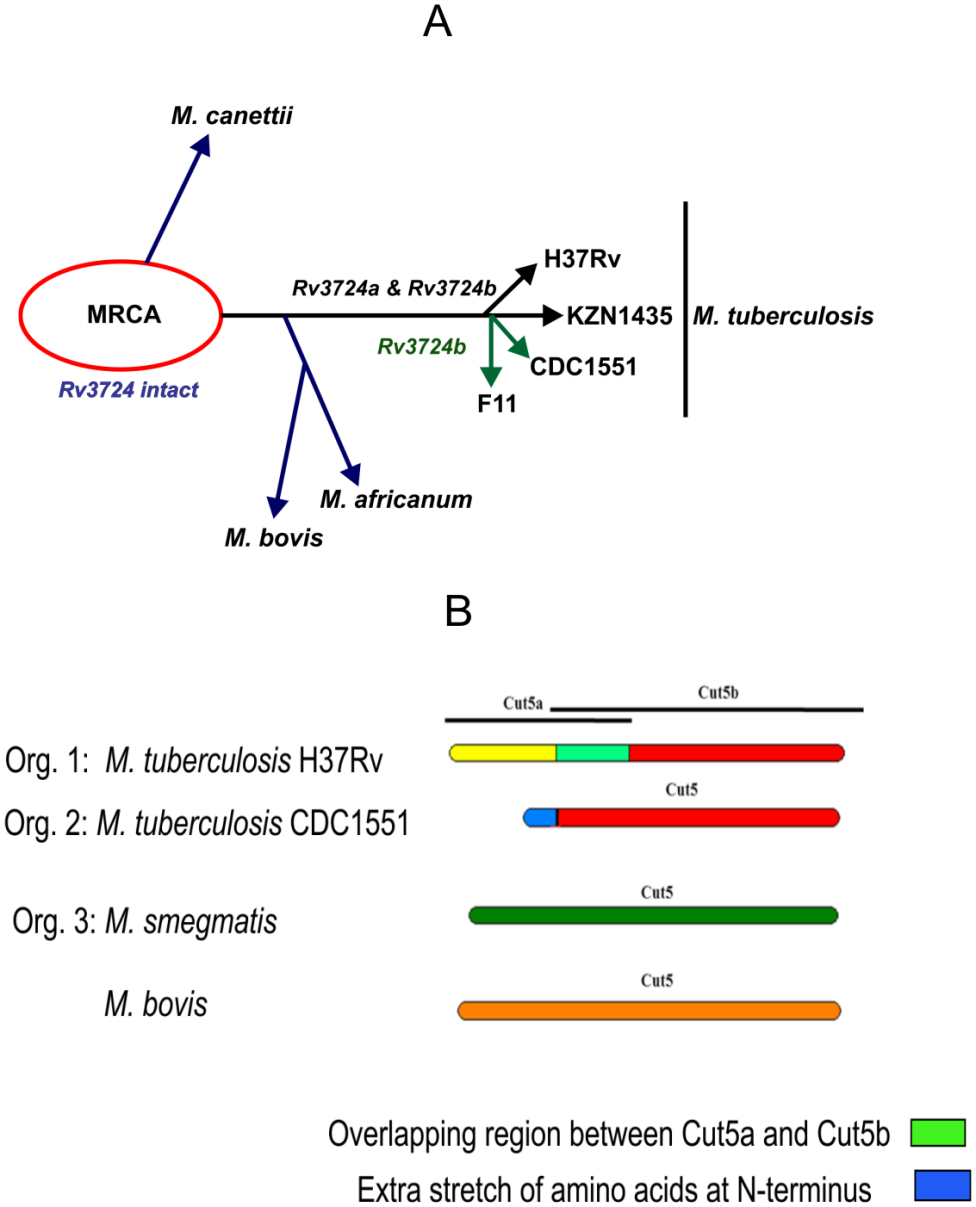
Proposed evolutionary pathway of *Rv3724a* (*cut5a*) and *Rv3724b* (*cut5b*) (Panel A) and possible organizations (Org. 1, 2 & 3) of Cut5 protein and its homologs (Panel B) in mycobacteria.

The unique genome arrangement of *cut5a* and *cut5b* and the presence of intact *cut5* in some species indicate that there is a special pattern of phylogeny among mycobacterial species and different strains of *Mtb* (Fig 8, panel A). The presence of both *cut5a* and *cut5b* homologs in *M*. *canettii*, *M*. *africanum* and *M*. *bovis* indicates the possibility of the existence of the same homolog in a most recent common ancestor (MRCA) as *M*. *canettii* got separated from the direct descendant of tubercle bacilli before the *M*. *africanum* and *M*. *bovis* lineage [28].

In order to check the distribution of *Mtb cut5b* and its homologs, mycobacteria species were divided into two groups: (i) human pathogenic *Mtb* strains CDC1551, F11, Harlem and KZN1435 and, (ii) other pathogenic species *M*. *canettii*, *M*. *africanum*, *M*. *marinum*, *M*. *ulcerans* and *M*. *avium*. Percentage identities of *cut5a* and *cut5b* were obtained using information available in the KEGG genome database. The three best identical homologs were included for analysis as depicted in Table 2. In order to study the occurrence of horizontal gene transfer events in *cut5* of *Mtb* the % GC contents of *cut5a* and *cut5b* were analyzed using the online available tool ‘GC Calculator’. Amino acid sequences of their homologs were retrieved from the KEGG database and analysis of these sequences revealed that *Mtb* H37Rv Cut5a and Cut5b were present in various virulent strains of *Mtb* as well as in other virulent mycobacterial species. Along with this, the *cut5 (Rv3724)* gene was found to have a unique genomic arrangement in different species and strains of mycobacteria. In a few strains of *Mtb*, annotated sequences indicated that two forms of the protein, Cut5a and Cut5b were possibly encoded by *Rv3724a* and *Rv3724b*. Similarly, in *Mtb* KZN1435 *MT_03768* and *MT_03769*, were assigned to encode homologs of Cut5a and Cut5b. Surprisingly, the products of both *MT_03768* and *MT_03769* were found to bear 100% identity with Cut5a and Cut5b, respectively. In other strains of *Mtb* i.e. F11 and CDC1551, homologs encoding Cut5b were present, but homologs encoding Cut5a were absent. Homologs of Cut5 from other human pathogenic species like *M*. *bovis*, *M*. *canettii* and *M*. *africanum* showed 99% identity with both Cut5a (Rv3724a) and Cut5b (Rv3724b). Homologs among non-mycobacterial species, members belonging to the class Actinobacteria such as *Rhodococcus sp*. RHAI, *Frankia*_Eud 1c, *Kineococcus radiotolerans*, *Gordonia brochialis* and *Nocardia farcinica*, were found to share 37–54% identity with *Mtb* H37Rv Cut5a and Cut5b proteins.

**Table 2:** Homologs of *M*. *tuberculosis* H37Rv Cut5a/Rv3724a and Cut5b/Rv3724b present in various mycobacterial and bacterial species.

Gene	Size	GC Content	Mycobacterial homologs		Non mycobacterial homologs
			Human pathogenic *M*. *tuberculosis* strains	Human pathogenic mycobacterial species	
***Rv3724a (cut5a)***	243 bp	64.6%	*Mtb* KZN1435; *TBMG_03768* [100%] [80/80]	*M*. *bovis*; *Mb_3751* [100%] [67/233]	*Kineococcus radiotolerance*; *Krad_4111* [53.2%] [62/294]
				*M*. *africanum*; *M&F_37330* [100%] [67/233]	*Segniliparus rotundus*; *Srot_0413* [47.3%] [74/278]
				*M*. *canettii*; *MCAN_37461* [97%] [67/233]	*Rhodococcus* sp. RHA1; *RHA1_RO00629* [37.5%] [60/247]
***Rv3724b (cut5b)***	564 bp	64.4%	*Mtb* CDC1551; *MT_3827* [99%] [187/207]	*M*. *bovis*; *Mb_3751* [100%] [166/233]	*Rhodococcus* sp. RHA1; *RHA1_ro00629* [54.8%] [168/247]
			*Mtb* KZN1435; *TBMG_03769* [99.5%] [187/207]	*M*. *africanum*; *M&F_37330* [100%] [166/233]	*Gordonia bronchalis*; *Gbro_4311* [44.8%] [174/239]
			*Mtb* F11; *TBFG_13756* [99.5%] [187/207]	*M*. *canettii*; *MCAN_37461* [95.8%] [166/233]	*Frankia*_Eul1c; *FraEul1c_0562* [40.7%] [167/226]

Names of the homologs in the table are mentioned as; specie; gene; % identity; amino acid overlap/total amino acids, e.g. *Kineococcus radiotolerance* **(specie)**; *Krad_4111* **(gene)**; (53.2%) **(% identity)**; [62/294] **(overlap/total amino acids)**

### *In silico* analysis of N- terminal sequence of *M*. *tuberculosis* H37Rv Cut5b and its homologs

Cut5b in human pathogenic *Mtb* strains: *Mtb* H37Rv, *Mtb* C, CDC1551, Haarlem, KZN1435 and F11 and other mycobacterial species: *M*. *bovis*, *M*. *marinum*, *M*. *ulcerans*, Agy99, *M*. *avium* K10, *Mycobacterium sp*. MCS, *Mycobacterium sp*. KMS, *Mycobacterium sp*. JLS and *M*. *smegmatis* vary in N-terminal sequence and protein length (S3 Fig, panel A & S4 Fig, panel B).

Based on the variation in N-terminal sequence and length of the protein, Cut5b homologs in various mycobacterial species were classified into three major categories (Fig 8, panel B);

1. Cut5b as part of a putative bipartite system: The genomic sequence of *Mtb* H37Rv encodes two different proteins: Cut5a (putative cutinase precursor) and Cut5b (putative cutinase) from a single gene. Surprisingly, such genomic arrangement, found in case of *Mtb* H37Rv, could only be seen in *Mtb* KZN1435, an MDR strain having a gene identical to *cut5a* which is present upstream of the *cut5b* homolog (S3 Fig, panel C and Table 2). In other mycobacterial species, homologs of *cut5a* are absent (S3 Fig, panel B).
2. Cut5b with an extra stretch of amino acids at an N-terminus: Extra stretch of highly charged 20 amino acids exist at the N-terminus of the protein in *Mtb* (S3 Fig, panel A). However, these stretches show no significant identity with Cut5a of *Mtb* H37Rv (S3 Fig, panel B), further emphasizing the unique genomic arrangement of *cut5* in *Mtb* H37Rv.
3. Cut5 as a single entity: In a few species like *M*. sp MCS, *M*. sp KMS and *M*. *smegmatis* etc., homologs of *cut5b* were predicted to encode a single protein having amino acid sequence variability ranging from 53 to 74%. Furthermore, N-terminus of this full length protein resembles neither that of Cut5a nor of Cut5b of *Mtb* H37Rv (S4 Fig, panel A & B).

## Discussion

*Mtb* has been reported to have the highest number of eukaryotic and prokaryotic interkingdom gene fusions of all the sequenced bacterial genomes [29]. Four mycobacterial cutinase genes, namely *Rv1758*, *Rv3451*, *Rv3452* and *Rv1984c* were thought to be acquired by horizontal gene transfer because no bacterial orthologues were found for mycobacterial cutinases. The coding regions of the mycobacterial genome have been affected by frame shifts owing to microsatellite InDels, which is an indication of gene fission/fusion, premature termination and length variation [30]. Genes affected by frame shift mutations encode for membrane proteins, cell wall synthesis proteins, transporters, PPE, PE_PGRS [31, 32] and hypothetical proteins. The ORF encoding Rv3724/Cut5 in *Mtb* H37Rv has been observed to split into two ORFs (*Rv3724a/cut5a* & *Rv3725b/cut5b*) due to the presence of a single nucleotide (T) insertion in the coding region which creates a stop codon in the coding region (Fig 1). These mutations are very common in *Mtb* and results in gene fission/fusion, premature termination and length variation. For instance, a single nucleotide deletion in the ORF, encoding functional isocitrate lyase, resulted in splitting of the ORF to produce two new ORFs (*Rv1915* & *Rv1916*) [30, 33]. The single nucleotide insertion in *Rv3724/cut5* does not seem to be the part of the core genome as the insertion is absent in homologs from other mycobacterial species. Since it is present only in *Mtb* strains (Fig 2) it can be speculated that this mutation has been acquired very recently.

It is reported that *Mtb* is devoid of a DNA mismatch repair system mediated by *mutS*, *mutL* and *mutH* genes [34, 35], rather plasticity to the genome is imparted through gene duplication and divergent evolution [10, 36]. Overlapping genes have unique evolutionary constraints, either to minimize genome size [37] or to elongate coding regions [38]. Moreover, RT-PCR analysis of *Rv3724/cut5* from *Mtb* H37Rv and its homolog *Mb_3751* from *M*. *bovis* BCG has demonstrated that *cut5* is transcribed as *cut5a* and *cut5b* only in *Mtb* H37Rv (Fig 3). However, the presence of *cut5a* transcript was not observed under the tested conditions. This may be due to the low copy number of its transcript or because of its condition specific transcription. Microarray analysis however revealed a four- fold excess of the *cut5a* transcript in dormant mycobacteria residing in lipid loaded macrophages [15].

Recombinant Cut5 of *M*. *bovis* appeared at ~24kDa in SDS-PAGE, similar to the size of recombinant Cut5b of *Mtb* H37Rv (Fig 4, panel A & B). However, the predicted size of *Mtb*rCut5b, based on molecular weight analysed by generunner (www.chembio.uoguelph.ca), and intact mass analysis was around 19 kDa and 20.9 kDa respectively (data not shown). Such reduced mobility in SDS-PAGE has been observed for other *Mtb* H37Rv cutinases Cut4 and Cut1 [8]. This may be due to post-translational modifications in the protein [39] or possible binding of different molar amounts of SDS with such proteins [8].

Previous studies [8, 13] and this study show that numbers of mycobacterial cutinases are capable of inducing a good antibody response. For instance, the recombinant proteins Cut2, Cut3, Cut6 and Cut7/Cfp21 elicited high titer antibodies (> 1:10000) [8, 13] while both *Mtb*Cut5b and *Mb*Cut5 sera showed similar titers (1:12,500) (data not shown). Furthermore, these antibody responses were quite specific to their respective proteins. *Mtb*Cut5b and *Mb*Cut5 sera reacted equally well with recombinant Cut5 of all the species tested i.e. *Mtb* H37Rv, *M*. *bovis* and *M*. *smegmatis* analysed by ELISA (data not shown) and western blotting (Fig 4, panels A & B). This was expected knowing the high levels of sequence homology among these proteins. Also, different levels of sequence homology (17–63%) result in the induction of low titer antibodies which are capable of cross-reacting with different cutinases [14]. However, no cross-reactivity was found between *Mtb* H37Rv cutinases (Fig 4, panel C). Taken together these results with previous reports [8, 13], it is likely that each cutinase has a unique immunodominant and specific epitope(s), besides sharing a few common epitopes. Further, cutinases Cut7/Cfp21, Cut2 and Cut6 have been shown to confer a moderate yet reproducible and significant level of protection in the murine model of an *Mtb* H37Rv infection [14]. Although the vaccine potential of *Mtb*rCut5b was not explored in this study, the observed antibody response, compared to those of the above cutinases, makes it a good candidate for further investigation into its protective (defensive) efficacy.

Attempts have been made earlier to localize various *Mtb* H37Rv cutinases. West *et al*., 2009 showed the presence of ~18kDa Cut2 and ~21kDa Cut7/cfp21 in culture filtrate, and ~32 kDa Cut6 in cell wall preparations by immunoblotting, while Cut4 has been reported to be localized in the cell wall of *Mtb* [40]. Reactivity of *Mtb*Cut5b sera in immunoblotting with cell extracts of *Mtb* H37Rv, *M*. *bovis* and *M*. *smegmatis* revealed that Cut5b and its homologs exist at ~24kDa in *Mtb* H37Rv and *M*. *bovis* and at ~27kDa in case of *M*. *smegmatis* (Fig 5, panel A). Such phenomena of the recognition of protein bands of different molecular size in different mycobacterial species has been previously noted in immunoblot analysis using *Mtb*Cut6 sera [7]. In few experiments, low molecular weight products e.g. ~22kDa, ~ 18kDa were also observed while checking the reactivity of *Mtb*Cut5b sera with the extract or membrane fraction (data not shown). It is likely that these lower bands were result of proteolytic degradation as reported for mycobacterial LprA [39]. In *Mtb* H37Rv, immunoelectron microscopy and immunoblotting revealed Cut5b to be associated with the cell wall, membrane and cytosol (Fig 5, panel B and Fig 6) under the given test conditions, indicating that like Cut6 & Cut4 it may have role in cell wall processes. Mycobacterial cutinases are classified under lipid metabolism enzymes and a role of some of these cutinases like Cut6 and Cut4 have been implicated in scavenging host lipids to provide a carbon source for the intracellular replicating mycobacteria [8, 27, 41]. Cut5b was found to be associated with the *Mtb* H37Rv cell wall at late exponential and stationary phases rather than in the early exponential phase (Fig 7) which may indicate that enzymes like cutinases involved in lipid metabolism can remain associated with the cell wall where they may be involved in cell wall modeling during intracellular growth and dormant phases [8].

According to Brosch et al., 2002, the most recent common ancestor of the tubercle bacilli (MRCA) resembles *Mtb* or *M*. *canettii* and the lineage of *Mtb* separated from the direct descendant of tubercle before the *M*. *africanum* and *M*. *bovis* lineage. All mycobacterial cutinases were found to have their homologs in *M*. *canettii*, *M africanum* and *M*. *bovis*, implying their presence in the common ancestor MRCA (Fig 8, panel A). Aberration in mean GC content has been used to study horizontal gene transfer (HGT) events in mycobacteria [42]. No observed difference between percentage GC content of *cut5a and cut5b* and mean GC content of mycobacteria i.e 65–66% (Table 2) indicated the absence of HGT events. Furthermore, no drastic changes could be seen in the sequences of these cutinases. Taken together, these observations suggest the ancient acquisition of these genes from *M*. *prototuberculosis* (MTBC) through vertical gene transfer.

Cutinases exist in both pathogenic and environmental strains of mycobacteria [9]. Analysis of *Rv3724/cut5* homologs from pathogenic and non-pathogenic strains of mycobacteria revealed its unique genomic arrangement (Table 2). In pathogenic mycobacterial species other than *Mtb*, like *M*. *bovis*, *M*. *africanum* and *M*. *canettii*, the homolog of *Rv3724/cut5* encoding for the full length protein product showed 99% identity with both Rv3724a/Cut5a and Rv3724b/Cut5b. Interestingly, there are some differences in the genomic arrangement of *Rv3724/cut5* within *Mtb* strains too. Unlike *Mtb* H37Rv, where two forms of the protein are encoded by the same gene *Rv3724/cut5*, *Mtb* KZN1435 has two different genes for encoding homologs of Cut5a and Cut5b (having 100% identity). Surprisingly, homologs of *Rv3724a/cut5a* were absent in *Mtb* F11 and CDC1551 but those of *Rv3724b/cut5b* were present. Thus, the presence of *cut5a* in *Mtb* KZN1435 may serve as a biomarker for this MDR strain and may be further exploited in studying other such strains as well.

Homologs of Cut5 were found to exhibit length variation, while the functional domains and catalytic triad remained conserved (S2 and S3 Figs). A peculiar 20 amino acid long N-terminus was observed in Cut5 homologues of strains belonging to *Mtb* except H37Rv (S4 Fig). It would be of interest to explore whether this has any functional significance. An *in silico* analysis revealed that the deleted N-terminal region of MT2802.1 possesses greater probability to act as a signal peptide suggesting that the protein is secreted. On the other hand, its longer counterparts in *Mtb* H37Rv and *M*. *bovis* possess negligible propensities of being signal peptides and thus are non-secretory. While in the case of mammalian cell entry (*mce*) family virulence proteins and some PPE proteins, length variation did not affect the function of the translated protein while keeping the functional domains well conserved [30].

In conclusion, this study demonstrates the existence of Cut5b in *Mtb* where it is present in the cell wall, membrane and cytosol. It has three possible organisations in different mycobacteria species. A single nucleotide (T) insertion in the *cut5* gene was found to be unique for *Mtb* strains. Studies are now required to further characterize Cut5 in order to evaluate its role in mycobacterial physiology and pathogenesis. Additionally, it is likely that the unique genomic arrangement in *cut5* can serve as a biomarker for differentiating *Mtb* strains from other mycobacteria species.

## Supporting Information

S1 Fig PCR amplification of *Rv3724/cut5*, *Rv3724a/cut5a* and *Rv3724b/cut5b* from *M*. *tuberculosis* H37Rv genomic DNA. Agarose gel (1%) showing *Rv3724/cut5*, *Rv3724a/cut5a* and *Rv3724b/cut5b* in lanes 1, 2 and 3 respectively, along with a 2log DNA ladder (NEB) in lane 4.
(TIF)

S2 Fig Multiple sequence alignment of Cut5 amino acid sequences from different mycobacterial species. Clustal X (1.81) was used for the alignment of Cut5 sequences of *M*. *tuberculosis* H37Rv (MtbRv), *M*. *tuberculosis* C (CG), *M*. *tuberculosis* CDC1551 (CDC), *M*. *tuberculosis* Haarlem (HG), *M*. *tuberculosis* KZN1435 (KZN), *M*. *tuberculosis* F11 (FG), *M*. *bovis* (bovis), *M*. *marinum* (marinum), *M*. *ulcerans* Agy99 (ulcerans), *M*. *avium* K10 (avium), *Mycobacterium* sp. MCS (MCS), *Mycobacterium* sp. KMS (KMS), *Mycobacterium* sp. JLS (JLS) and *M*. *smegmati*s (smeg).
(TIF)

S3 Fig Amino acid sequence alignment of *Mtb* H37Rv Cut5a and Cut5b with pathogenic *M*.*tuberculosis* strains. Clustal X (1.81) was used for the alignment of Cut5 sequences of *M*. *tuberculosis* H37Rv (MtbRv), *M*. *tuberculosis* C (CG), *M*. *tuberculosis* CDC1551 (CDC), *M*. *tuberculosis* Haarlem (HG), *M*. *tuberculosis* KZN1435 (KZN), *M*. *tuberculosis* F11 (FG). Panel A: Alignment of *Mtb*Cut5b with the Cut5 of virulent species mentioned above shows the presence of N-terminal 20 amino acid conserved sequence (depicted in box) in virulent strains of *M*. *tuberculosis*. Panel B: Alignment of *Mtb*Cut5a with Cut5 of human pathogenic strains of *M*. *tuberculosis*. Panel C; Clustal omega alignment showing 100% identity among Cut5a of *M*. *tb* H37Rv and *M*. *tb* KZN1435. Gaps introduced by Clustal, to optimize the alignment, are indicated by ‘-’. Identical amino acids are represented by asterisk (*), conserved residues by colon (:) and semi conserved residues by dot (.). Lower bar graphs represent the homology between the aligned sequences.
(TIF)

S4 Fig Amino acid sequence alignment of Cut5 from *Mycobacterium* sps. other than *M*. *tuberculosis*. Clustal X (1.81) was used for the alignment of Cut5 sequences of *M*. *marinum* (marinum), *M*. *ulcerans* Agy99 (ulcerans), *M*. *avium* K10 (avium), *Mycobacterium* sp. MCS (MCS), *Mycobacterium* sp. KMS (KMS), *Mycobacterium* sp. JLS (JLS) and *M*. *smegmati*s (smeg). Panel A: Alignment of *Mtb*Cut5b with Cut5 of the mycobacterial species mentioned above. Panel B: Alignment of *Mtb*Cut5a with Cut5 of pathogenic species mentioned above. Gaps introduced by Clustal, to optimize the alignment, are indicated by ‘-’. Identical amino acids are represented by asterisk (*), conserved residues by colon (:) and semi conserved residues by dot (.). Lower bar graphs represent the homology between the aligned sequences.
(TIF)
