# Supplementary figures and images for: Heterogeneity among Homologs of Cutinase-Like Protein Cut5 in Mycobacteria
Source: PLoS One. 2015 Jul 15;10(7):e0133186. doi: 10.1371/journal.pone.0133186 (PMC4503659; doi:10.1371/journal.pone.0133186)

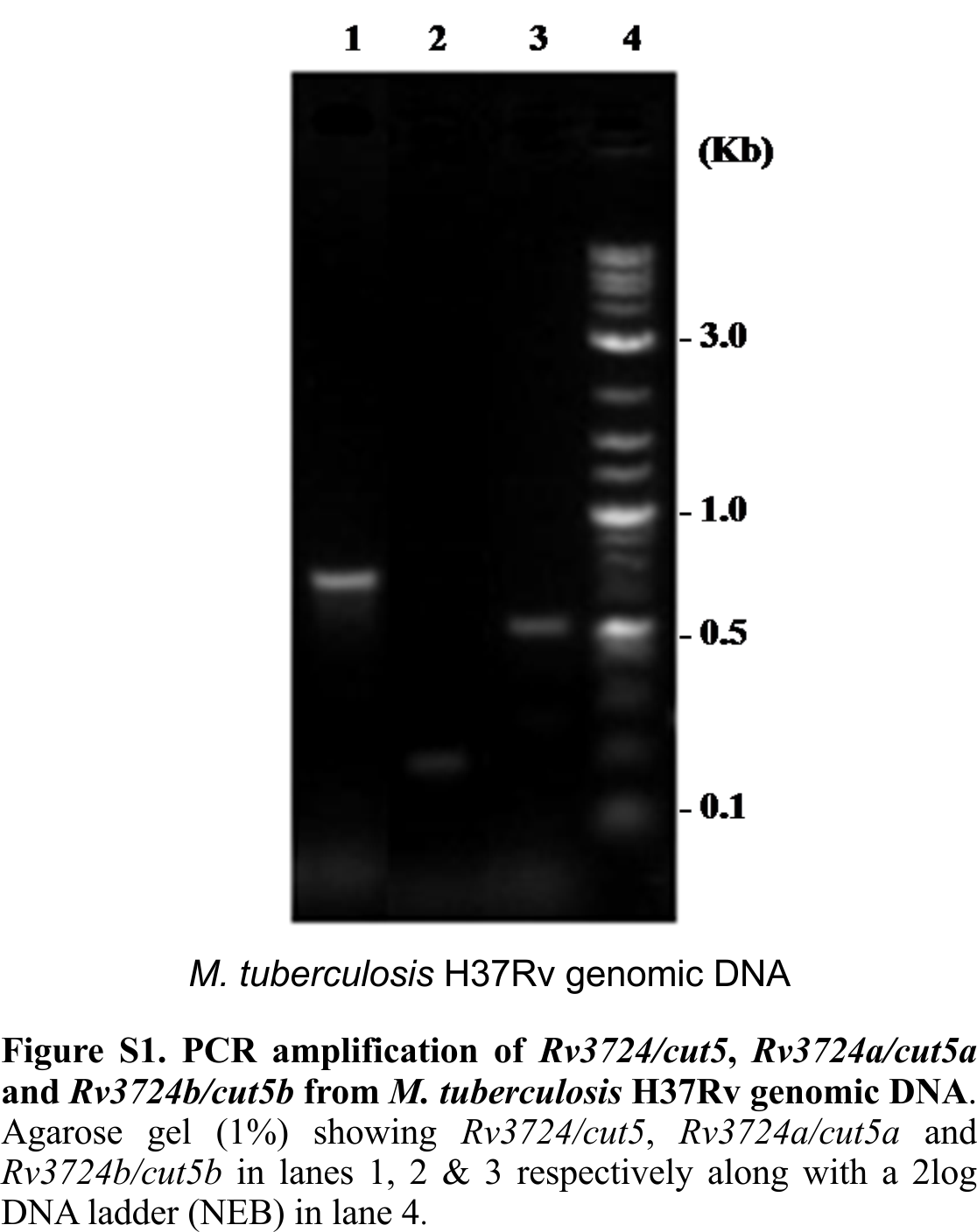

Supplement: S1 Fig — Agarose gel (1%) showing Rv3724/cut5, Rv3724a/cut5a and Rv3724b/cut5b in lanes 1, 2 and 3 respectively, along with a 2log DNA ladder (NEB) in lane 4. (TIF) [file pone.0133186.s001.tif]

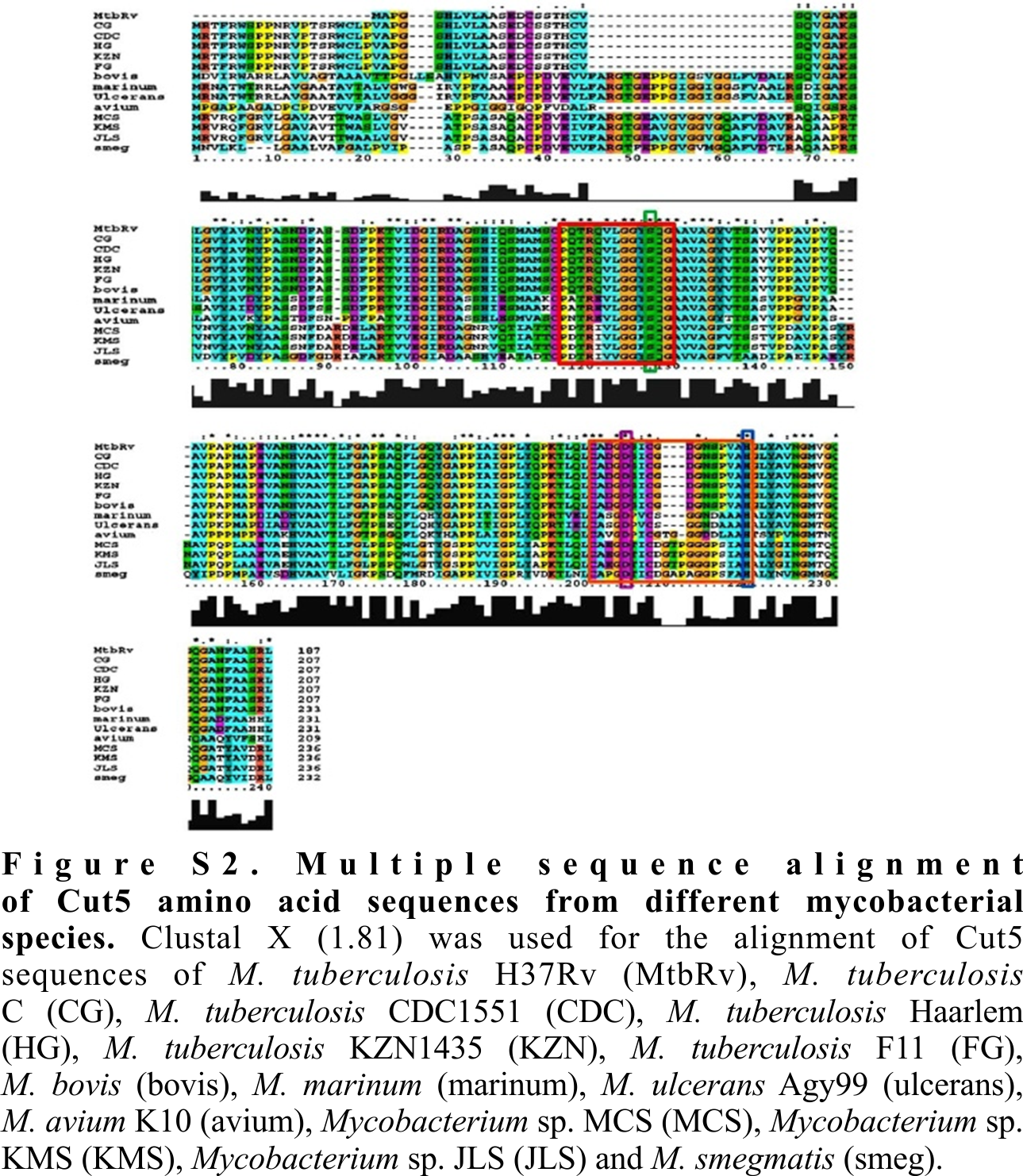

Supplement: S2 Fig — Clustal X (1.81) was used for the alignment of Cut5 sequences of M. tuberculosis H37Rv (MtbRv), M. tuberculosis C (CG), M. tuberculosis CDC1551 (CDC), M. tuberculosis Haarlem (HG), M. tuberculosis KZN1435 (KZN), M. tuberculosis F11 (FG), M. bovis (bovis), M. marinum (marinum), M. ulcerans Agy99 (ulcerans), M. avium K10 (avium), Mycobacterium sp. MCS (MCS), Mycobacterium sp. KMS (KMS), Mycobacterium sp. JLS (JLS) and M. smegmatis (smeg). (TIF) [file pone.0133186.s002.tif]

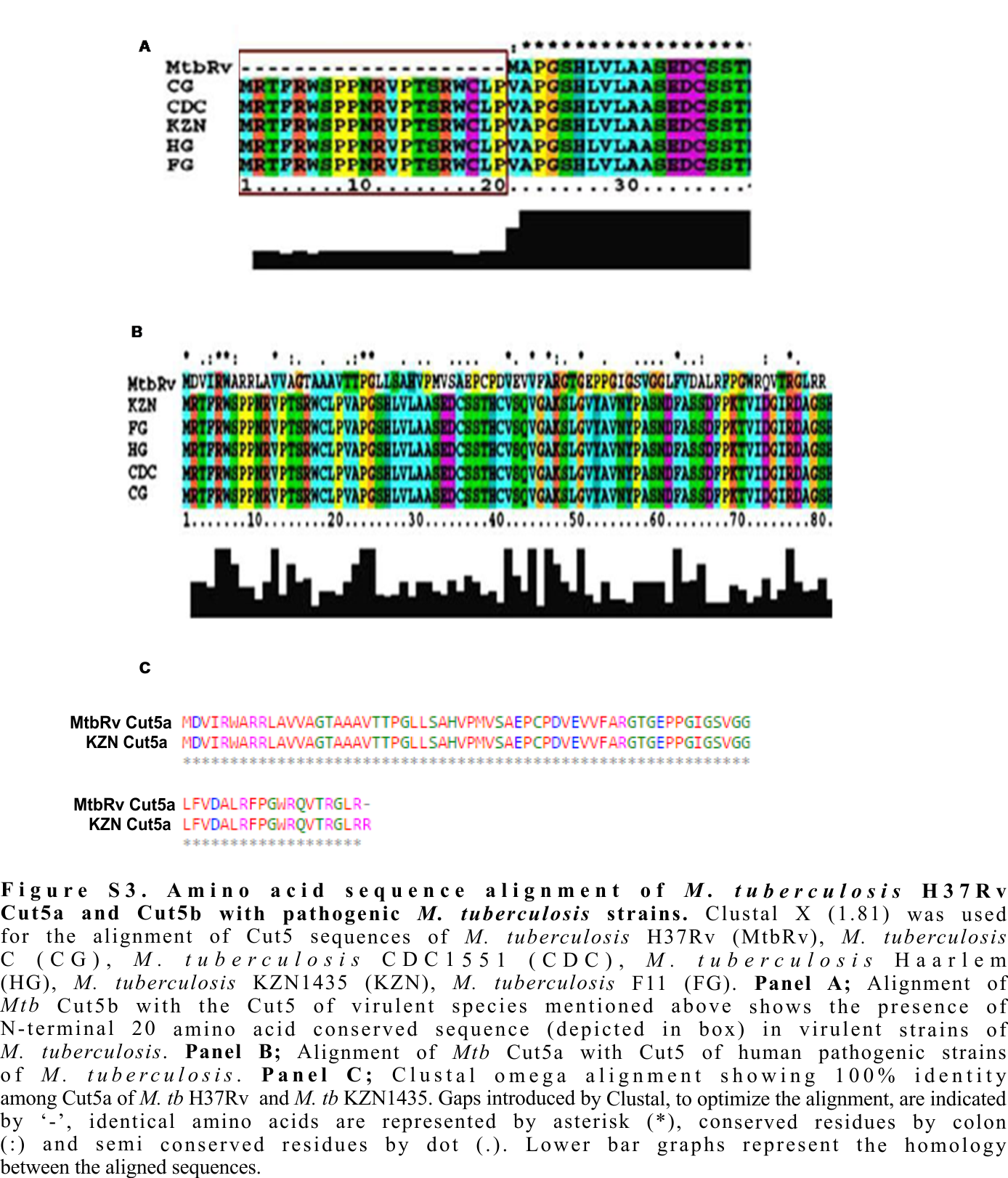

Supplement: S3 Fig — Clustal X (1.81) was used for the alignment of Cut5 sequences of M. tuberculosis H37Rv (MtbRv), M. tuberculosis C (CG), M. tuberculosis CDC1551 (CDC), M. tuberculosis Haarlem (HG), M. tuberculosis KZN1435 (KZN), M. tuberculosis F11 (FG). Panel A: Alignment of MtbCut5b with the Cut5 of virulent species mentioned above shows the presence of N-terminal 20 amino acid conserved sequence (depicted in box) in virulent strains of M. tuberculosis. Panel B: Alignment of MtbCut5a with Cut5 of human pathogenic strains of M. tuberculosis. Panel C; Clustal omega alignment showing 100% identity among Cut5a of M. tb H37Rv and M. tb KZN1435. Gaps introduced by Clustal, to optimize the alignment, are indicated by ‘-’. Identical amino acids are represented by asterisk (*), conserved residues by colon (:) and semi conserved residues by dot (.). Lower bar graphs represent the homology between the aligned sequences. (TIF) [file pone.0133186.s003.tif]

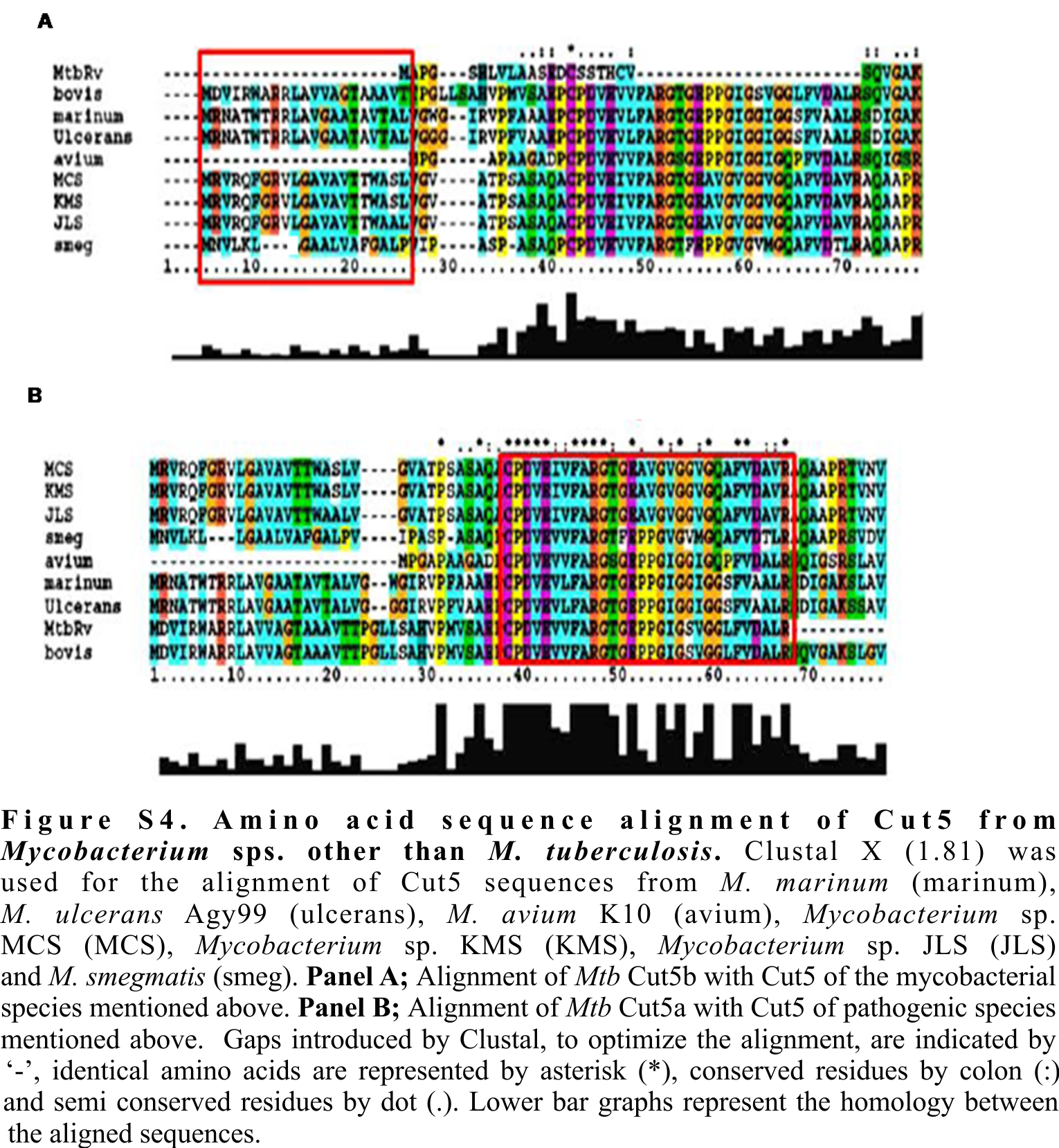

Supplement: S4 Fig — Clustal X (1.81) was used for the alignment of Cut5 sequences of M. marinum (marinum), M. ulcerans Agy99 (ulcerans), M. avium K10 (avium), Mycobacterium sp. MCS (MCS), Mycobacterium sp. KMS (KMS), Mycobacterium sp. JLS (JLS) and M. smegmatis (smeg). Panel A: Alignment of MtbCut5b with Cut5 of the mycobacterial species mentioned above. Panel B: Alignment of MtbCut5a with Cut5 of pathogenic species mentioned above. Gaps introduced by Clustal, to optimize the alignment, are indicated by ‘-’. Identical amino acids are represented by asterisk (*), conserved residues by colon (:) and semi conserved residues by dot (.). Lower bar graphs represent the homology between the aligned sequences. (TIF) [file pone.0133186.s004.tif]
